# Regulating Craving by Anticipating Positive and Negative Outcomes: A Multivariate Pattern Analysis and Network Connectivity Approach

**DOI:** 10.3389/fnbeh.2018.00297

**Published:** 2018-12-04

**Authors:** Johann D. Kruschwitz, Vera U. Ludwig, Lea Waller, David List, David Wisniewski, Uta Wolfensteller, Thomas Goschke, Henrik Walter

**Affiliations:** ^1^Division of Mind and Brain Research, Department of Psychiatry and Psychotherapy CCM, Charité—Universitätsmedizin Berlin, Corporate Member of Freie Universität Berlin, Humboldt—Universität zu Berlin, and Berlin Institute of Health, Berlin, Germany; ^2^Collaborative Research Centre 940 “Volition and Cognitive Control”, Technische Universität Dresden, Dresden, Germany; ^3^Berlin School of Mind and Brain, Humboldt-Universitaet zu Berlin, Berlin, Germany; ^4^Mindfulness Center, Brown School of Public Health, Brown University, Providence, RI, United States; ^5^Department of Experimental Psychology, Ghent University, Ghent, Belgium; ^6^Faculty of Psychology, Technische Universität Dresden, Dresden, Germany

**Keywords:** anticipated emotions, self-control, volition, future thinking, fMRI, self-regulation, craving

## Abstract

During self-control, we may resist short-term temptations in order to reach a favorable future (e.g., resisting cake to stay healthy). The neural basis of self-control is typically attributed to “cold,” unemotional cognitive control mechanisms which inhibit affect-related regions via the prefrontal cortex (PFC). Here, we investigate the neural underpinnings of regulating cravings by mentally evoking the positive consequences of resisting a temptation (e.g., being healthy) as opposed to evoking the negative consequences of giving in to a temptation (e.g., becoming overweight). It is conceivable that when using these types of strategies, regions associated with emotional processing [e.g., striatum, ventromedial prefrontal cortex (vmPFC)] are involved in addition to control-related prefrontal and parietal regions. Thirty-one participants saw pictures of unhealthy snacks in the fMRI scanner and, depending on the trial, regulated their craving by thinking of the positive consequences of resisting, or the negative consequences of not resisting. In a control condition, they anticipated the pleasure of eating and thus, allowed the craving to occur (now-condition). In line with previous studies, we found activation of a cognitive control network during self-regulation. In the negative future thinking condition, the insula was more active than in the positive condition, while there were no activations that were stronger in the positive (> negative) future thinking condition. However, additionally, multivariate pattern analysis showed that during craving regulation, information about the valence of anticipated emotions was present in the vmPFC, the posterior cingulate cortex (PCC) and the insula. Moreover, a network including vmPFC and PCC showed higher connectivity during the positive (> negative) future thinking condition. Since these regions are often associated with affective processing, these findings suggest that “hot,” affective processes may, at least in certain circumstances, play a role in self-control.

## Introduction

In our daily lives, we are often confronted with situations requiring self-control, which can be defined as the ability to resist temptation and override impulsive responses in order to behave consistently with our long-term goals and social norms (Baumeister et al., 2007; Hassin et al., 2010; Hofmann et al., 2012). For example, we may resist eating tasty desserts in order to stay slim, or perform daily workouts to stay fit and attractive. In cognitive neuroscience, the neural basis of self-control is often attributed to “cold” cognitive control mechanisms of the prefrontal cortex (PFC) that compete with “hot” affective impulses (e.g., McClure et al., 2004), or modulate value signals (Hare et al., 2009, 2011). Similarly, Heatherton and Wagner (2011) emphasize that “self-regulatory failure occurs whenever the balance is tipped in favor of subcortical areas” (p. 1) associated with impulses and emotions as opposed to control-related prefrontal regions.

While such dual-system models are useful, they might have limitations: human decisions requiring self-control may not solely be explained by “cold” cognitive processes alone, but may also depend on the mobilization of emotions consistent with long-term outcomes (see Phelps et al., 2014; Lerner et al., 2015 for a review). That is, conflicts may not only involve a struggle between reason and emotion, but also a struggle between different emotions associated with short-term and long-term outcomes of decisions. In line with this, Berkman et al. (2017) have proposed that self-control situations can be framed as instances of value-based decision-making with different values assigned to the short-term and the long-term option (see also Bulley et al., 2016).

Specifically, theories of affective forecasting (e.g., Loewenstein et al., 2001; Mellers and McGraw, 2001; Gilbert and Wilson, 2007) suggest that decision-making and self-control are crucially influenced by anticipated emotions. Thinking about the future may evoke emotional representations of long-term outcomes, and these may support the assignment of value to options (Pezzulo and Rigoli, 2011). For example, thinking about long-term costs of not eating healthily (“I will gain weight”) may evoke negative emotions, whereas thinking about benefits of abstaining from unhealthy food (“I will stay healthy”) may evoke positive emotions. In line with this, anticipated emotions have been shown to influence decision-making and goal-directed behavior (Mellers and McGraw, 2001; Perugini and Bagozzi, 2001; Baumgartner et al., 2008; Patrick et al., 2009; Pezzulo and Rigoli, 2011). The differences of being motivated by potential gains as opposed to losses has also been described and studied, but usually in the context of monetary rewards (e.g., Kahneman and Tversky, 1979; Paschke et al., 2015). There are hardly any neuroimaging studies that have investigated this topic for primary rewards (e.g., food), and for more complex potential gains and losses (e.g., being fit vs. putting on weight).

In typical food craving-regulation studies, participants are told to decrease their craving or to cognitively reappraise short-term rewards by thinking of the* negative* consequences of giving in to the temptation (e.g., Siep et al., 2012; Giuliani and Pfeifer, 2015; Cosme et al., 2018). For example, Kober et al. (2010) showed pictures of cigarettes and food items to 21 smokers. They then asked them to regulate their craving by thinking of the (negative) long-term consequences of smoking or eating the food. Likewise, Hollmann et al. (2011) (p. 2) asked participants “to reinterpret the subjective value of unhealthy food with respect to long-term consequences (that is, negative effects on health and social life).” When comparing regulating with allowing craving, studies typically find activation in a network previously implicated in cognitive control and emotion regulation, including the dorsolateral PFC (dlPFC; Kober et al., 2010; Hollmann et al., 2011; Giuliani et al., 2014; Giuliani and Pfeifer, 2015; Cosme et al., 2018). It is puzzling why neuroimaging researchers have been focusing so much on *negatively* oriented reappraisal strategies for the regulation of cravings.

There was only one neuroimaging study, to our knowledge, that included a self-regulation condition for food in which participants anticipated the *positive* consequences of resisting (Yokum and Stice, 2013). The authors asked participants to regulate craving using, depending on the trial, a positive or a negative future thinking strategy (i.e., benefits of not eating vs. costs of eating). Again, during regulation, there was activation of regions typically implicated in higher cognitive processes, namely the superior frontal gyrus and the ventrolateral PFC (vlPFC). There were no significant differences between the negative and positive future thinking strategy. However, the sample in this study consisted of 21 adolescents with a mean age of 15 years and, in this study, only a traditional univariate analysis approach was used to analyze the data.

Relevant to the current study, using more abstract stimuli, Kruschwitz et al. (2018) measured brain activation while participants anticipated bi-valent stimuli: aversive sounds that were presented simultaneously with monetary gains. Crucially, during the anticipation of the bi-valent stimulus (in Experiment 2), participants either focused on the positive aspect of the outcome (the monetary gain) or the negative aspect (the aversive sound). Focusing on the negative was associated with increased activation of the bilateral insula during anticipation, whereas focusing on the positive was associated with increased activation in the ventral striatum (VS), ventromedial PFC (vmPFC) and posterior cingular cortex (PCC; see also Doré et al., 2017). Moreover, activation in the insula correlated with the degree to which participants experienced negative emotions (distress) during anticipation, while activation in the VS, vmPFC and PCC correlated with the degree to which participants experienced positive emotions (pleasure and relief; the former for all three regions and the latter only for VS and vmPFC). The logical next question is whether the same neural mechanisms can be observed in a more complex self-control situation during the regulation of food cravings.

Here, we set out to delineate the neural differences between positive and negative future thinking in the regulation of food craving in an adult sample, with a focus on regions of the brain that have previously been associated with affective processing. In contrast to Yokum and Stice (2013), we did not only use a standard univariate approach to analyze our data, but combined it with a more sensitive multivariate pattern analysis approach (MVPA; Haynes and Rees, 2006) and a network connectivity analysis. While traditional univariate analyses simply determine the overall activation of each individual voxel, MVPA is highly sensitive in detecting differences in neural activation patterns across voxels (Davis et al., 2014). In the MVPA analysis, we used searchlight decoding to answer the question “Where in the brain does the activity pattern contain information about the experimental condition?” (i.e., positive vs. negative future thinking; Kriegeskorte et al., 2006; p. 3863). A network-based task-related functional connectivity approach (Rissman et al., 2004; Zalesky et al., 2010) was also used, to investigate the differences in connectivity patterns between positively and negatively oriented regulation strategies. This extends previous studies that mostly focused on connectivity between single brain regions (e.g., Kober et al., 2010), by using a more holistic network-based approach.

In line with previous studies, we expected to replicate the finding of a cognitive control network, including the lateral PFC, during the use of both reappraisal strategies, in the univariate analysis. We further hypothesized that the negative vs. positive regulation strategy would be associated with differential activation in regions previously associated with emotional processing and anticipated emotions: the VS, vmPFC, amygdala, insula and PCC (Bechara and Damasio, 2005; Sharot et al., 2007; D’Argembeau et al., 2008; Knutson and Greer, 2008; Bray et al., 2010; Benoit et al., 2014; Lin et al., 2015). These were the same regions of interest (ROIs) as used by Kruschwitz et al. (2018). In line with Kruschwitz et al. (2018), for the univariate analysis, we expected higher activation of the insula for the negatively oriented control strategy, and higher activation of VS, vmPFC and PCC for the positively oriented strategy. In addition or alternatively to this, we predicted differential patterns of neural activation in these regions in the positive vs. negative condition, as revealed by multivariate analysis. Moreover, we expected there to be changes in connectivity patterns between the regions depending on whether a positively or negatively oriented strategy was used. More specifically, regions classically associated with positive affect processing (i.e., vmPFC, VS, PCC) might show enhanced connectivity during the positive future thinking condition, while regions usually associated with negative affect processing (i.e., insula, amygdala) might show enhanced connectivity during the negative condition.

## Materials and Methods

### Participants

Data from 31 healthy, non-obese participants were analyzed (16 female, 15 male; mean age: 25.9 ± 3.34 years; range: 18–32). One additional participant was excluded from data analysis because we observed abnormalities in cortical development that could have compromised statistical analyses. On average, participants had a score of 6.35 (SD 2.61) on the *Restraint Scale* for assessing restrained eating behavior (Herman and Polivy, 1980; German version by Dinkel et al., 2005), which is in the normal range; thus, participants were eating normally. Eleven out of the 31 participants said they regularly engaged in sports (the other 20 did not), and the average score for drinking alcohol in the sample was 2.77 (SD: 0.669) on a scale of 5 (from 0: never to 5: often). None of the participants reported a lifetime history of psychiatric disorder. Additional exclusion criteria included pregnancy and other general MRI contraindications. Written informed consent was provided by all participants and the study was approved by the Ethics Committee of Technische Universität Dresden. After completing the fMRI tasks, participants were debriefed and received compensation.

### Design

Participants were asked not to eat for 2 h before coming to the lab. Prior to the experiment, participants rated their craving in response to each snack on a 5-point Likert scale to establish a baseline. The stimuli were 36 photographic images of unhealthy snacks, which were selected based on their ability to induce craving and validated in a previous study (Ludwig et al., 2014).

Next, participants read standardized instructions for the experiment (included in the Supplementary Material). Specifically, they were instructed to regulate their craving by thinking about either the negative long-term consequences of repeatedly consuming the snacks or the positive consequences of abstaining from the presented snacks. To understand the instructions better, a list of example strategies was provided to participants that could be used for downregulating craving (e.g., negative: “I will become obese” or “I will develop dental problems,” and positive: “I will look good at the beach” or “I will feel fit and energized;” see Supplementary Material for the entire list). The experimenter went through the examples with participants and explained the concepts of negative and positive future thinking. However, participants were also free to choose their own strategies, which felt most relevant to them. In a control condition we asked participants to not regulate, but to anticipate the rewarding nature of the stimulus itself (now-condition). This was done in a within-subject design with all participants applying all strategies.

The experimenter verified that participants understood and were able to apply the strategies during the experiment. To control for low-level visual confounds in the neuroimaging data that would compromise multivariate decoding, each strategy was indicated by two distinct visual cues (see Wisniewski et al., 2015 for a similar approach). Memorization of the cues was verified by a stringent quiz, and participants performed 12 training trials of the regulation task on a different set of snacks (see Supplementary Material). The scanning session began once the experimenter verified that participants had performed the task correctly during the training.

**Figure 1:**
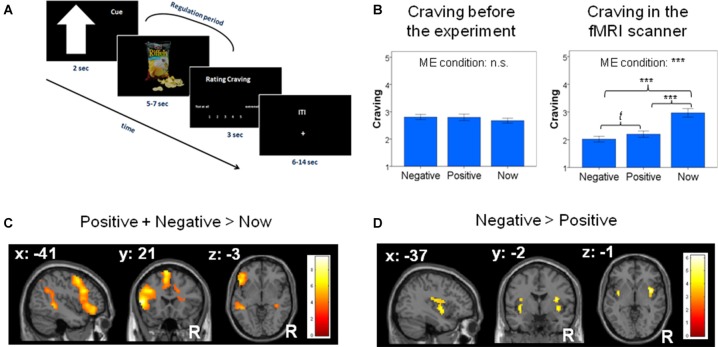
**(A)** Trial sequence with cues indicating to regulate the craving by thinking about either the negative long-term consequences of repeatedly consuming the snacks (e.g., “I will become obese”) or the positive consequences of abstaining from the presented snacks (e.g., “I will look good at the beach”). In a control condition we asked participants to not regulate, but to anticipate the rewarding nature of the stimulus itself (“now”-condition). **(B)** Left: snacks assigned to each condition did not differ in terms of elicited craving at baseline. Right: craving was significantly regulated by the use of (future) thinking strategies in the fMRI scanner. ME: ANOVA main effect. n.s.: not significant, t: trend, ***: *p* < 0.001. Error bars depict 95% confidence intervals of the mean, adapted for within-subject designs (Cousineau, 2005). **(C)** Whole brain analysis for the contrast “positive + negative > now” revealed significant activation [all *p* < 0.05 family wise error (FWE)-corrected] of the cognitive regulation network. **(D)** Region of interest (ROI) analyses for the contrast “negative > positive” future thinking revealed significant activation in the insula (*p* < 0.05 small-volume FWE-corrected; activation displayed at *p* < 0.001 uncorrected for illustrative purposes).

During scanning, participants completed a cognitive emotion regulation task that extends the self-control study by Kober et al. (2010); (Figure 1A). In each trial, we first instructed participants to use one of the valence-specific self-control strategies using the respective abstract visual cue (2 s). After the cue, an image of a snack was presented for 6 ± 1 s, during which the participants applied the strategy. After each trial, participants rated their craving for the snack on a 5-point Likert scale by moving a cursor using response keys. The rating scale was presented for up to 3 s or until the participant had given a response. Each trial concluded with an inter-trial interval of 10 ± 4 s, during which participants saw a fixation cross (Figure 1C). Participants completed five runs consisting of 36 trials each (12 trials per condition, one for each snack) that were presented in a pseudorandomized order. The same 12 snacks (six sweet and six salty) were always presented for the same strategy within one participant, as the strategies were expected to become associated with the specific snack types. Across participants, the assignment of the snacks to the three strategies was permutated to exclude bias produced by the specific stimuli.

### Experimental, Statistical and fMRI Data Analysis Software

The experiment was implemented using E-prime software (Version 2.0). Behavioral data analyses were performed with IBM SPSS Statistics 21. ROIs were created using FSLView Version 3.2.0 (FMRIB centre, University of Oxford). Standard univariate fMRI data analyses for all experiments were performed with SPM8^1^. Multivariate pattern analysis was carried out using the Decoding Toolbox (Hebart et al., 2015). Network-based task-related functional connectivity analyses were performed using BASCO (Göttlich et al., 2015) and GraphVar (Kruschwitz et al., 2015).

### Functional Magnetic Resonance Imaging

Participants were scanned in a 3T Siemens Tim Trio MRI scanner with a 12-channel head coil. We acquired 380 images for each of the five runs with 33 axial slices in descending order using a T2*-sensitive one-shot gradient-echo echo-planar imaging (EPI) sequence with the following parameters: repetition time 2,000 ms, echo time 30 ms, flip angle 78°, field of view 192 mm, matrix size 64 × 64, voxel size 3 × 3 × 3 mm and inter-slice spacing 0.75 mm.

Moreover, we acquired a high-resolution image for spatial referencing with 192 sagittal slices using a T1-weighted magnetization-prepared rapid gradient-echo (MPRAGE) sequence with the following parameters: repetition time 1,900 ms, inversion time 900 ms, echo time 2.52 ms, flip angle 9°, matrix size 256 × 256 and voxel size 1 × 1 × 1 mm.

### Analysis of Behavioral Data

We first made sure that the snacks assigned to each of the three strategies did not differ in terms of craving values prior to the experiment (i.e., without any application of a strategy). For this, we carried out a repeated-measures ANOVA with the within-subject factor “assigned strategy” (3 levels) on the craving values obtained before the fMRI experiment.

For the main analysis, for each participant, we calculated the mean of the craving ratings per condition (including 60 trials in total per condition; 12 trials in each of the five runs) during the fMRI experiment and applied a repeated-measures ANOVA to the resulting values (i.e., one within-subject factor “condition” with 3 levels, *n* = 31). We then calculated dependent *t*-tests for the *post hoc* comparisons of interest (positive vs. now, negative vs. now, and positive vs. negative) and applied Bonferroni correction for three comparisons.

### fMRI Analysis

#### Regions of Interest (ROIs)

ROIs for the univariate and multivariate pattern analysis were the vmPFC, bilateral VS (defined as nucleus accumbens), bilateral insula, bilateral amygdala and bilateral PCC. Previous studies have identified these regions as being involved in future thinking and anticipated emotions (Sharot et al., 2007; D’Argembeau et al., 2008; Knutson and Greer, 2008; Staudinger et al., 2009; Bray et al., 2010; Sharot et al., 2010; Benoit et al., 2011; Carlson et al., 2011; Winecoff et al., 2013; Benoit et al., 2014; Greenberg et al., 2015; Lin et al., 2015; Kruschwitz et al., 2018). ROIs were derived from the Harvard-Oxford Structural Atlas. The vmPFC ROI was taken from a previous study using the same stimuli (Ludwig et al., 2014) in which a probabilistic ROI was constructed. The basis for this ROI were coordinates of vmPFC activation of previous studies on value-based decision making (see Supplementary Material for details).

For the network connectivity analysis, the aforementioned ROIs were considered to be too big, as they had the purpose to restrict the univariate and multivariate analysis to these regions on a rougher scale and to correct for family-wise error (FWE) within these regions. Averaging voxel time courses over these big regions for the connectivity analysis would not be reasonable. Therefore, for network construction we instead chose the 116 AAL ROIS (Tzourio-Mazoyer et al., 2002) over the Harvard-Oxford Structural Atlas (69 ROIs) and the aforementioned vmPFC ROI due to its finer granularity (resulting in a larger network and potentially higher number of observable interactions) but comparable neuroanatomical spanning range.

#### Preprocessing

All volumes were realigned (i.e., the runs were first realigned to each other, by aligning the first scan of each run to the first scan of the first run. Then the images within each run were aligned to the first image of the run) and slice-time corrected in a standard way (reference slice 16). Volumes were normalized (unified segmentation of T1-image, 3 mm isotropic voxels) to the MNI (Montreal neurological institute) template (ICBM 152) and smoothed with a Gaussian kernel with a full-width half-maximum (FWHM) of 8 mm. Of note, for MVPA analyses all data were processed in native space (i.e., the resulting accuracy maps were normalized and smoothed (FWHM: 8 mm) after MVPA computations for second level analyses; see below).

#### Analysis

We then constructed a general linear model (GLM) for each participant. To correct for motion-related effects, we included six motion regressors in the GLM. All regressors were convolved with the SPM8 canonical hemodynamic response function. Intrinsic autocorrelations were modeled by a first-order autoregressive model. Low frequency oscillations were removed with a high-pass filter with a cut-off frequency at 1/128 Hz.

The analysis then consisted of three independent parts. First, we analyzed the data using a standard univariate approach. For this, on the first level, cues and image presentation were combined and modeled by a separate regressor for each strategy that lasted 2 + 6 ± 1 s. The rating period after the image presentation and the inter-trial intervals were modeled as two separate regressors of no interest. We then calculated the contrasts of interests regarding the cognitive control network (positive + negative > now), and valence-dependent anticipation processes (negative > positive; positive > negative) in each participant and, on the second level, carried out one-sample *t*-tests to assess activations and significance on the group level. To ensure the validity of the results derived by the directed contrasts, we additionally (*post hoc*) calculated an F-test spanning all three conditions in a flexible factorial design which included subject as a factor. Masking the respective t-contrasts with the derived F-Test search space resulted in the same set of activations.

Second, we performed MVPA to overcome constraints of univariate analyses and to localize brain regions associated with differential processing of anticipated emotions. For the MVPA, we again constructed a GLM for each participant as before. However, we constructed one regressor for each strategy and cue combination lasting 2 + 6 ± 1 s, which resulted in two regressors per strategy (i.e., 2 strategies × 2 cues per strategy = 4 regressors).

Although ROI-based decoding (e.g., Gallivan et al., 2011; Chadwick et al., 2012) is often used to determine if brain regions contain information discriminating experimental conditions, we decided to use a searchlight decoding approach (Kriegeskorte et al., 2006). This approach makes no spatial *a priori* assumptions about functional regions (the ROIs are only used at the end of the analysis to constrain the area being looking at and to correct for multiple comparisons). Using searchlight decoding, it is possible to detect if only a specific part of a ROI contains information of interest. Specifically, we applied multivariate pattern classification for each subject using a support vector classifier (SVC) with a linear kernel and a fixed regularization parameter (*C* = 1) on the parameter estimates of the GLM (Cox and Savoy, 2003; Mitchell et al., 2004; Kamitani and Tong, 2005; Haynes and Rees, 2006), as implemented in LIBSVM^2^). Here, we constructed a sphere with a radius of three voxels around each voxel in the acquired volumes and extracted parameter estimates for the two regulation conditions (i.e., two cues per condition = 4 extractions) for each of the N voxels in the respective sphere, resulting in an N-dimensional pattern vector. A SVC was subsequently trained to discriminate pattern vectors of the conditions “cue 1 strategy 1” vs. “cue 1 strategy 2.” The classification performance was then tested using the independent pattern vectors of “cue 2 strategy 1” vs. “cue 2 strategy 2” (i.e., evaluation of the classifier performance via cross-classification on the independent strategy cues which also controls for potential problems of overfitting). We used a leave one-run out cross-validation scheme with our five runs (i.e., we trained on four runs and used the 5th one for testing; and repeated this five times in total with all possible combinations). We then calculated the mean prediction accuracy across the cross-classification steps and assigned this value to the central voxel of the sphere. The classification was repeated for every sphere in the volume, resulting in a three-dimensional accuracy map for each subject.

The resulting accuracy maps were normalized and smoothed as before. For group-level analyses, the accuracy maps of each subject were entered in one-sample *t*-tests against chance level (50%). As the use of theoretically derived chance levels (e.g., 50% chance with two classes) in multivariate pattern analyses has been recently criticized (Combrisson and Jerbi, 2015), we additionally performed permutation testing to derive voxel-wise empirical chance levels for our data (see Supplementary Material).

To account for interindividual differences in classification accuracies between conditions due to differential success in applying the two strategies (see “Behavioral Results” Section), we entered the latter as a covariate in group-level analyses (mean rating positive − mean rating negative) in both univariate and MVPA analyses. For both univariate and MVPA analyses, significance was assessed as follows: for ROI-based as well as whole-brain analyses we applied a FWE-corrected statistical threshold of *p* < 0.05 (peak-level), which has been shown to be an appropriate correction method (Eklund et al., 2016). For ROI-based fMRI analyses no cluster-extent threshold was used in addition to the (FWE-corrected, small volume correction; SVC) voxel peak-level thresholding.

Third, we performed a network-based task-related functional connectivity approach (Rissman et al., 2004; Zalesky et al., 2010) to investigate if affect-related subnetworks would be differentially engaged during self-control. By focusing on network-wide connectivity changes between task-conditions, this network analysis approach overcomes a-priori restrictions of previous studies that focused on interactions between single brain regions (e.g., Kober et al., 2010). Specifically, we used a beta-series correlation analysis approach to establish task-related functional connectivity between brain regions (Rissman et al., 2004) for the two regulation conditions (i.e., positive and negative). For network construction we chose the 116 AAL ROIS (Tzourio-Mazoyer et al., 2002), as explained above. Based on this parcellation, we extracted the mean beta-series for each experimental condition (across runs) and derived a connectivity matrix (116 × 116) for each experimental condition (positive and negative regulation) per subject by correlating all ROI beta-series with each other applying Pearson’s linear correlation. Subsequently, for identification of a subnetwork associated with connectivity changes between the positive and the negative regulation condition (i.e., graph component), we applied the network-based statistic (NBS) approach (Zalesky et al., 2010) as implemented in GraphVar (Kruschwitz et al., 2015) in which we entered both connectivity matrices per subject in a repeated measures GLM. To estimate the null-distribution of maximal graph component size (i.e., to control the FWE rate of the graph component), we used a permutation-based non-parametric approach with 1,000 random permutations. To derive sets of supra-threshold links (i.e., the effect associated subnetwork) we set the initial-link threshold to *p* < 0.001 (permutation-based single-link significance). Graph components were considered significant with *p* < 0.05 FWE-corrected. Additionally, in a less conservative approach, we used a link-based approach to explore individual connections between any ROI pair within the network that may ultimately form an extended connected component. Here, we used a link-wise false discovery rate correction procedure (corrected *p* < 0.001) that, as compared to NBS, may provide additional information on focal effects concerning individual connections.

## Results

### Behavioral Results

There were no significant differences in the pre-experimental craving values between the snacks assigned to each strategy (*F*_(2,60)_ = 1.434, *p* = 0.246; see Figure 1B, left), suggesting that the random assignment of the snacks to the categories had been successful.

In the main analysis of the craving values measured during the fMRI experiment, we found a significant main effect of strategy: *F*_(1.686,50.566)_ = 45.158, *p* < 0.001; Huynh-Feldt correction was applied due to a violation of the assumption of sphericity). As Figure 1B (right) shows, craving values during a negative-thinking strategy and a positive-thinking strategy were both significantly lower than when focusing on the now (neg. vs. now: (*T*_(30)_ = 8.721, *p* < 0.0001; pos. vs. now (*T*_(30)_ = 6.319, *p* < 0.0001). The negative-thinking strategy tended to be associated with lower craving than the positive thinking strategy (*T*_(30)_ = 2.369, *p* = 0.024) but this was not significant after applying Bonferroni-correction (required significance value when conducting three tests: 0.017).

### fMRI Results

Whole-brain analyses for the contrast *positive + negative > now* revealed significant activation in the cognitive regulation network, specifically the left dlPFC, left vlPFC and left middle temporal gyrus (MTG; *p* < 0.05; Figure 1C; Table 1A). As shown in the Supplementary Material, analyses for the contrasts negative > now and positive > now revealed a similar and comparable activity of the well-established cognitive regulation network in both cognitive control strategies.

**Table 1A:** Results of standard univariate whole-brain analyses with voxel-wise one-sample *t*-test for the contrast “positive + negative > now” (*n* = 31; all *p*: FWE, whole-brain corrected).

Whole-brain	L/R	Cluster size	Peak-voxel activity			*T*	*Z*	*p*—peak voxel FWE
			*x*	*y*	*z*			
dlPFC	L	1721	−36	5	49	6.74	5.25	<0.01
vlPFC	L		−54	23	7	9.59	6.49	<0.01
SFG	L	447	−6	17	64	7.78	5.75	<0.01
MTG	L	544	−42	−40	−2	5.76	4.71	0.01

*dlPFC, dorsolateral prefrontal cortex; vlPFC, ventrolateral prefrontal cortex; SFG, superior frontal gyrus; MTG, middle temporal gyrus; FWE, family wise error corrected*.

ROI-based analyses for the contrast *negative > positive* revealed significant activations in the bilateral insula (*p* < 0.05; Figure 1D; Table 1B). Whole-brain analysis did not reveal any additional activations. ROI and whole-brain analyses for the *positive > negative* contrast did not reveal any significant activations.

**Table 1B:** Results of standard univariate region of interest (ROI) analyses with voxel-wise one-sample *t*-test for the contrasts “negative > positive” future thinking (*n* = 31; all *p*: FWE, small-volume corrected).

ROI (bilateral)	L/R	Cluster size	Peak-voxel activity			*T*	*Z*	*p—*peak voxel FWE
			*x*	*y*	*z*			
negative > positive							
insula	L	89	−36	−1	−2	4.48	3.90	0.02
	R	157	39	5	−5	6.18	4.95	<0.01

*FWE, family wise error corrected*.

**Figure 2:**
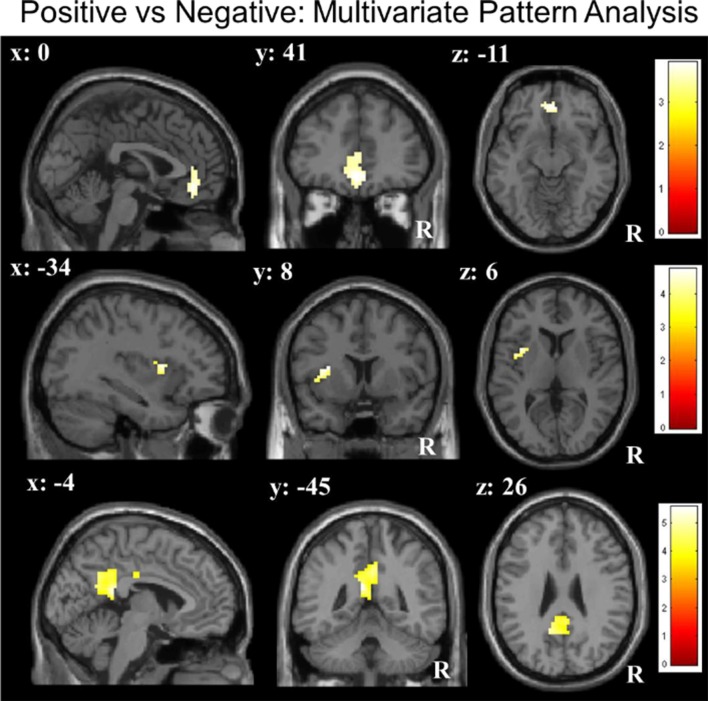
Multivariate pattern analyses (searchlight decoding) within ROIs for the contrast “negative vs. positive” future thinking revealed significant discriminatory information (all *p* < 0.05 small-volume FWE-corrected) in the ventromedial prefrontal cortex (vmPFC), insula and posterior cingulate cortex (PCC).

ROI-constricted analyses of MVPA accuracy maps revealed a significant discrimination between the positive and negative conditions in the vmPFC (55.78%; *Z* = 3.51, *p* < 0.05), left insula (55.75%; *Z* = 3.97, *p* < 0.05) and PCC (55.77%; *Z* = 4.28, *p* < 0.05; Table 1C; Figure 2). A whole-brain analysis additionally revealed a significant discrimination in the bilateral visual cortex. When performing the same analyses with the permutation derived empirical chance level maps, we additionally observed a significant discrimination between the positive and negative conditions in the right anterior insula and VS (see Supplementary Material).

**Table 1C:** Results of multivariate pattern analyses (searchlight decoding) within ROIs with voxel-wise one-sample *t*-test (against chance level) for the contrast “negative vs. positive” future thinking (*n* = 31; all *p*: FWE, small-volume corrected).

ROI (bilateral)	L/R	Cluster size	Peak-voxel activity			*T*	*Z*	*p—*peak voxel FWE
			*x*	*y*	*z*			
positive vs. negative							
vmPFC	-	84	0	41	−11	3.96	3.51	0.045
PCC	L	207	−6	−46	19	5.11	4.28	<0.01
insula	L	40	−30	8	13	4.62	3.97	0.02

*vmPFC, ventromedial prefrontal cortex; PCC, posterior cingulate cortex; FWE, family wise error corrected*.

**Figure 3:**
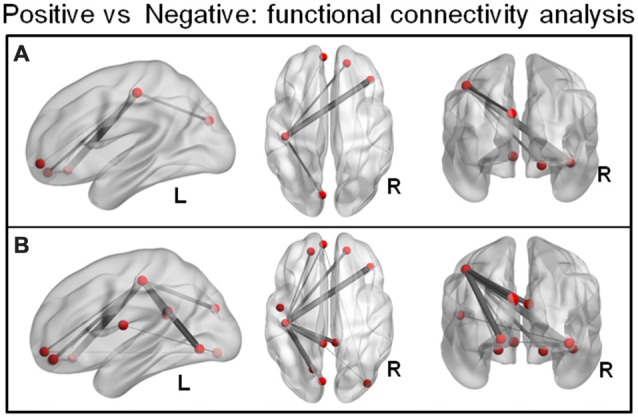
**(A)** Network-based statistic (NBS) on the condition-specific task-related functional connectivity matrices (positive vs. negative) revealed a sub-network (*p* = 0.005, FWE) comprising five interconnected brain areas that showed stronger connectivity during the positive compared to the negative regulation condition. **(B)** A less conservative link-based FDR correction approach (corrected *p* > 0.001) resulted in an extended component of 11 regions (entailing the NBS detected network).

NBS on the condition-specific task-related functional connectivity matrices (positive vs. negative) revealed a single subnetwork (*p* = 0.005) comprising five interconnected brain areas. These areas included the left cuneus, left post central gyrus, right superior orbitofrontal gyrus, right inferior orbitofrontal gyrus and left medial orbitofrontal gyrus (Figure 3A; the area in the VMPFC was similar to the region identified in the MVPA: medial orbitofrontal cortex). The less conservative link-based FDR correction approach resulted in a sub-network of 11 regions (covering the NBS-detected component) but additionally included the right inferior occipital gyrus, left lingual gyrus, left and right PCC, left rolandic operculum and left superior orbitofrontal gyrus (Figure 3B). This (extended) network was characterized by stronger connectivity during the positive vs. the negative regulation condition. There was no network that showed stronger connectivity during the negative compared to the positive regulation condition.

## Discussion

Motivated by theories of affective forecasting and the assumption that human behavior may be influenced by anticipated emotions, we performed an fMRI experiment to investigate the role of brain regions associated with affect regulation during craving regulation. Using MVPA and a network connectivity analysis, we investigated the neural correlates of a strategy of anticipating positive consequences of resisting temptations (e.g., staying fit) as compared with anticipating negative consequences of succumbing to temptations (e.g., becoming overweight).

While self-control is often seen as a “cold” higher cognitive process which modulates “hot” affective processes from top-down, we report evidence suggesting that self-control may actually involve “hot” affective processes as well. Specifically, we find that regions implicated in emotional processing show differential activation and connectivity patterns when participants use the two strategies (positive vs. negative future thinking). Thus, such cognitive control strategies may not only involve purely cognitive representations of outcomes, but may actually involve anticipated emotions which then influence decision-making (see also Damasio, 1994).

More specifically, in a univariate pattern analysis, we found insula activation in the negative future thinking (> positive) strategy, but, contrary to our expectation, no activation in the vmPFC, PCC, or VS in the positive (> negative) condition. Using a more sensitive decoding approach, however, we observed that the two strategies were indeed associated with differential activation in the anterior insula, vmPFC and PCC (as well as, using a more explorative statistical threshold, the VS), regions previously associated with affect and valence processing (e.g., Kruschwitz et al., 2018). Using a network analysis approach based on task-related functional connectivity (Rissman et al., 2004; Zalesky et al., 2010), we further found that positive future thinking (> negative) was associated with stronger connectivity of a network involving these regions (specifically those linked with positive affective processing), including among other areas orbitofrontal regions (similar to the one found in the multivariate analysis) and the PCC.

Previous studies found that the same regions (i.e., vmPFC and dorsal PCC) appeared to encode the subjective value of delayed monetary rewards in intertemporal choice tasks (Kable and Glimcher, 2007; Peters and Büchel, 2009, 2010; Benoit et al., 2011; Liu et al., 2012), and also correlated with taste ratings, health ratings and overall value of food items at decision time (Hare et al., 2011). Furthermore, vmPFC has been shown to be involved in the computation and encoding of subjective and emotional value during imagined scenarios (Benoit et al., 2014; Lin et al., 2015) and during emotion regulation (Winecoff et al., 2013). Combining these findings, we can speculate that these regions are implicated in processing future positive affect. Thus, it is possible that anticipated affect may be crucial for the determination of the reward value of anticipated outcomes (Pezzulo and Rigoli, 2011). The same may be true for the insula as it has been implicated in the anticipation of negative emotion (Berns, 2006; Knutson and Greer, 2008; Carlson et al., 2011) and was speculated to influence the computation of value signals (e.g., Hare et al., 2010).

Of course it has to be kept in mind that the regions that we term affect-related are not* exclusively* linked with emotions but also with other processes (e.g., self-referential processing, or the default mode network; e.g., Northoff et al., 2006; Uddin et al., 2009). However, there is a bulk of studies showing a link between these regions and affective processing (e.g., Bechara and Damasio, 2005; Sharot et al., 2007; Knutson and Greer, 2008), and in a previous study (Kruschwitz et al., 2018), we found that activation in the exact ROIs used here correlated with anticipated affect. The current findings challenge dual systems views of self-control, because the cognitive control system was found to be activated alongside with involvement of brain regions associated with affect. Previously, affect-related brain regions were thought to only be inhibited during self-control.

A surprising finding was that the visual cortex also showed differential activation in the two conditions. We can speculate that this is due to the two strategies involving relatively different visual imagination processes. Also unexpectedly, we did not detect any significant effects in the amygdala. This may be due to the fact that negative future thinking does not induce very high level of arousals, as for example loud bursts of noise do, which relate to amygdala activation (see Davis and Whalen, 2000; Carlson et al., 2011).

Note that we did not collect subjective ratings of emotions during scanning because such ratings (in addition to the craving ratings) could have interfered with the task and thereby influenced neural activation. In an unpublished study, we found that participants find it difficult to subjectively distinguish between craving (“How strong is my craving while I imagine something in the future?”) vs. feeling (“How do I feel while I imagine this?”). In this unpublished study, we found almost perfect correlations between these two concepts. Future studies could train participants more extensively in differentiating between these experiences to reassess this issue. Such studies may also investigate if the strength of the anticipated affect (e.g., in a self-report/behavioral study) predicts the degree to which craving is successfully regulated.

There are two points to note with regard to the snack stimuli. First, the same snacks were used for the same strategies across the experiment within participants. This was done since we expected that snacks would automatically get associated with a certain strategy. Across participants, however, the assignment of snacks to the three strategies was permutated to exclude bias produced by specific stimuli. Second, the food stimuli in the current study were craved by this sample to an intermediate rather than a high degree, with a craving value of around 3 out of 5 on the Likert scale for the NOW-condition. Giuliani et al. (2013) showed that regulation strategies have stronger effects when foods are highly craved (in their study: a craving value of 3.7 out of 5) when compared to regulating craving for food that is craved very little (in their study: 2.3 out of 5). However, regulation strategies were still effective, albeit to a lesser degree, for the stimuli that were craved little in their study. To maximize effects in future studies, however, we recommended to ask participants not to eat before the experiment for more hours than we did (e.g., 3 or 4 instead of 2 h), in order to increase craving further (e.g., Hare et al., 2009).

The present findings are consistent with theories of affective forecasting that assume the prominence of anticipated emotions (e.g., Mellers and McGraw, 2001; Loewenstein et al., 2001; Gilbert and Wilson, 2007) and their presumed influence on decision-making and goal-directed behavior (Mellers and McGraw, 2001; Perugini and Bagozzi, 2001; Baumgartner et al., 2008; Patrick et al., 2009; Pezzulo and Rigoli, 2011). Thus, they may provide neural evidence for the standing assumption that volitional future thinking may evoke affective anticipations of long-term outcomes that could support self-control in the balancing of short-term and long-term costs and benefits (Pezzulo and Rigoli, 2011; Benoit et al., 2014).

Thus, self-control may not only rest on cognitive representations of future outcomes but may simultaneously rely on anticipated emotions via affective feeling states. Future studies should assess this further by using additional dependent variables to measure affective experience.

## Author Contributions

JK was involved in the conception/design of the work, the acquisition, analysis, interpretation of data, and in writing the article. VL was involved in the analysis and interpretation, partly in the study preparation (stimuli) and in writing the article. UW, HW and TG were involved in the conception/design of the work and in interpreting the results. LW and DW were involved in conceptualizing the analysis and carrying it out. All authors revised the work critically for important intellectual content.

## Conflict of Interest Statement

HW received a speaker honorarium of Servier. The remaining authors declare that the research was conducted in the absence of any commercial or financial relationships that could be construed as a potential conflict of interest.

## Footnotes

^1^http://www.fil.ion.ucl.ac.uk/spm

^2^https://www.csie.ntu.edu.tw/~cjlin/libsvm/
