# Supplementary material for: Regulating Craving by Anticipating Positive and Negative Outcomes: A Multivariate Pattern Analysis and Network Connectivity Approach
Source: Front Behav Neurosci. 2018 Dec 4;12:297. doi: 10.3389/fnbeh.2018.00297 (PMC6289042; doi:10.3389/fnbeh.2018.00297)
Supplement: Supplementary file 1 [file Data_Sheet_1.PDF]

Supplementary material

Standardized instructions, shown on a screen (translated from German)
The instructions consisted of four pages, shown on a black screen. After participants had seen these four pages, there was a quiz in which participants were asked which symbols were associated with which strategies. The quiz was only completed after participants had given the correct response 10 times in succession per symbol.

Page 1:
The following runs will prepare you for the experiment in the MRI scanner.
This study is about regulating craving for the snacks that we have just shown you. Now we will present three strategies to you. Using the first two strategies, you are supposed to regulate your craving for the snacks. The third strategy involves that you imagine what it would be like to eat the snack.
Every strategy is coded by two symbols. In the scanner, these symbols will be used as cues for the strategy that you are supposed to use. Remember them well!
You have as much time as you need to memorise the strategies on the following pages. Please press enter to continue!

Page 2:
Strategy 1: Positive consequences
Think about the positive consequences of not eating the snack.
The experimenter will provide a list of examples of positive consequences.
This strategy is coded by the following symbols:

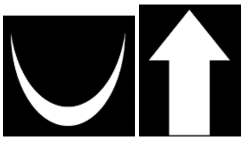
(shown on black background on the instructions screen)

Page 3:
Strategy 2: Negative consequences
Think about the negative consequences of eating the snack.
The experimenter will provide a list of examples of negative consequences.
This strategy is coded by the following symbols:

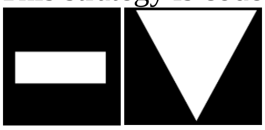
(shown on black background on the instructions screen)

Page 4:
Strategy 3: Now
Think about what it would be like to eat the snack now.
This strategy is coded by the following symbols:

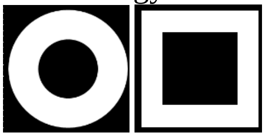
(shown on black background on the instructions screen)

List of examples of positive and negative consequences (translated from German)
These lists of examples were provided to participants to offer ideas for strategies (see above). Participants were, however, free to choose their own strategy (i.e., a strategy that was most relevant to them). The lists were based on strategies described by students in a small survey conducted via email, and included the strategies that were most often described by the students.

<u>Positive consequences</u>
If I eat healthily,
… I will, on average, live 6 years longer.
… I will be a role model for my peers.
… I will feel fit and energized.
… I will look good at the beach.
… my figure will stay attractive.
… I will have myself under control.
… I will have cleaner skin.
… my diet will be more varied.

<u>Negative consequences</u>
If I don't eat healthily,
… I will feel sick.
… I will lose the overview and eat too much.
… I will be obese.
… I will harm the environment.
… I will develop dental problems.

vmPFC ROI definition - methods
The vmPFC ROI (510 voxels) was taken from a previous study using the same stimuli (Ludwig et al. 2014) in which a probabilistic ROI was constructed based on the coordinates of vmPFC activation of several previous studies on value based decision making. Specifically, we extracted the peak coordinates in rACC and/or mOFC from fMRI publications which investigated the brain correlates of the subjective value for food items (Chib et al. 2009; Hare et al. 2009; Hare et al. 2011; Litt et al. 2011; Plassmann et al. 2007; Plassmann et al. 2010). We then created the ROI by the following three steps (Schubert et al. 2008): (1) We estimated the probability that a voxel at a given position showed neural activity as reported in the corresponding literature by calculating a 3D normal (Gaussian) distribution G(x, y, z) as follows (Turkeltaub et al. 2002):

$$G(x,y,z)=\frac{1}{2\pi\sqrt{|Det(C)|}}\exp\left(-\frac{1}{2}\begin{bmatrix}x-\bar{x} & y-\bar{y} & z-\bar{z}\end{bmatrix}\right)C^{-1}\begin{bmatrix}x-\bar{x}\\ y-\bar{y}\\ z-\bar{z}\end{bmatrix}$$

where C is the covariance matrix for all coordinate triples x, y, z from the underlying literature and x, y, z are the mean values of the x, y, and z coordinates, respectively. (2) We defined the outer limits of the final ROI by a threshold of 2 standard deviations of the resulting 3D distribution. (3) We created a binary mask including all voxels within these spatial boundaries.

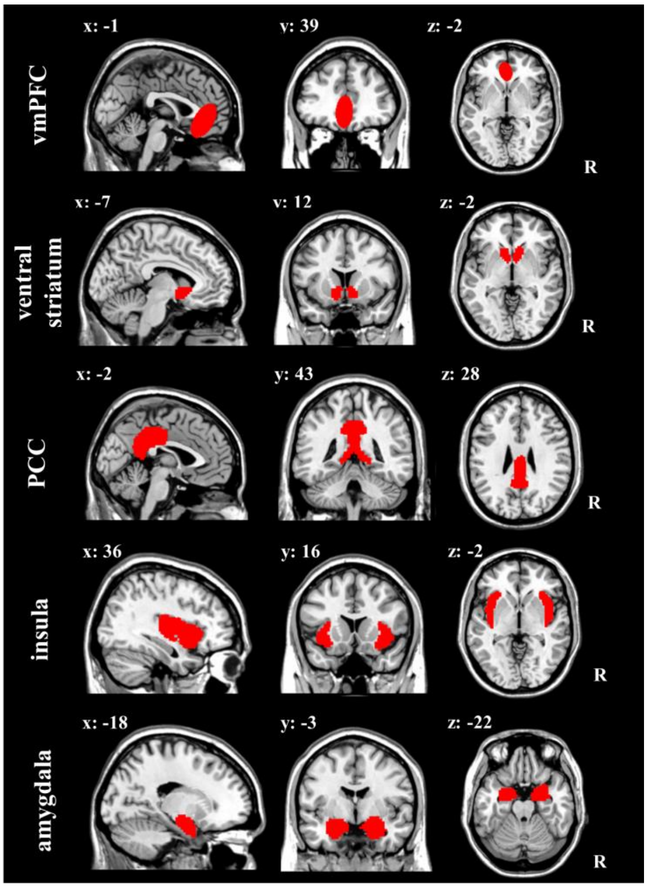

Suppl. Figure 1:
Regions of interest (ROIs) were the vmPFC, bilateral ventral striatum (defined as nucleus accumbens), bilateral insula, bilateral amygdala, and bilateral posterior cingulate cortex (PCC). ROIs were derived from the Harvard-Oxford Structural Atlas (bilateral insula: 2043 voxels; bilateral amygdala: 725 voxels; bilateral ventral striatum: 248 voxels; bilateral PCC: 982 voxels). The vmPFC ROI (510 voxels) was taken from a previous study (Ludwig et al. 2014).

Cognitive regulation network for each strategy separately

Whole-brain analyses for both contrasts *negative* > *now* and *positive* > *now* revealed significant activation in the cognitive regulation network, specifically the left dorsolateral prefrontal cortex, left ventrolateral prefrontal cortex and left middle temporal gyrus (the cluster in the middle temporal gyrus did not reach significance in the positive > now comparison) (p<.05 FWE-peak level; Table 2; Suppl. Figure 1).

Suppl. Table 1. Standard univariate whole-brain analyses with voxel-wise one-sample t-test for the contrast 'negative > now' and 'positive > now' (n=31; all p: FWE, whole-brain corrected).

Whole-brain	L/R	Cluster size	Peak-voxel activity			*p* – peak voxel FWE
			x	y	z	
negative > now						
dlPFC	L	1268	-36	5	49	<0.01
vlPFC	L		-54	23	7	<0.01
SFG	L	399	-6	17	64	<0.01
MTG	L	419	-45	-40	-2	0.01
positive > now						
dlPFC	L	1050	-39	5	52	<0.01
vlPFC	L		-54	23	7	<0.01
SFG	L	348	-6	17	67	<0.01
MTG	L	214	-42	-40	-2	n.s.

dlPFC: dorsolateral prefrontal cortex; vlPFC: ventrolateral prefrontal cortex; SFG: superior frontal gyrus; MTG: middle temporal gyrus; n.s.: not significant

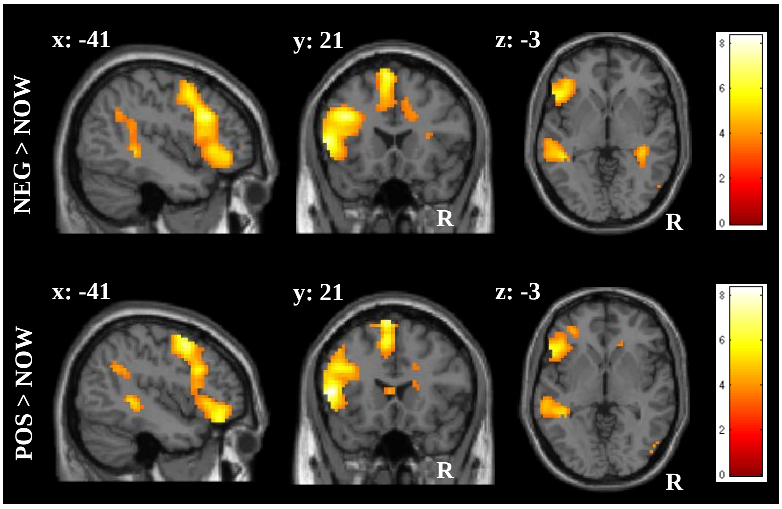

Suppl. Figure 2: Standard univariate whole-brain analyses with voxel-wise one-sample t-test for the contrast 'negative > now' and 'positive > now' (p<.05 FWE-peak level) (activation displayed at p<.001 uncorrected for illustrative purposes).

MVPA with empirical chance levels - methods
As the use of theoretically derived chance levels (e.g., 50% chance with 2 classes) in multivariate pattern analyses has recently been criticized (Combrisson & Jerbi, 2015), we additionally performed permutation testing to derive voxel-wise empirical chance levels for our data. Specifically, we performed a leave-one run out cross-validation approach in which we permuted the respective labels of the extracted parameter estimates in the training runs (i.e., 8 labels per condition = 16 labels in 4 runs) and tested the classification performance with the original label assignments in the remaining test run (i.e., 5 repetitions with the possible combination of training and testing, calculation of mean prediction accuracy across the cross-validation steps, assignment of this value to the central voxel of the sphere, and repetition for every sphere in the volume). This cross-validation procedure was repeated 1000 times per subject, which resulted in 1000 three-dimensional random permutation accuracy maps for each subject. To derive the empirical chance level of classification accuracy, we subsequently computed the mean decoding accuracy across the 1000 permutations per voxel, which resulted in a voxel-wise chance level map per subject. We then subtracted this map from the non-permuted cross-validation accuracy map per subject, which was subsequently normalized and smoothed as before and entered in a one-sample t-test (against empirical chance level) for group analysis. Significance was assessed as follows: we applied an FWE-corrected statistical threshold of $p < 0.05$ (peak-level), which has been shown to be an appropriate correction method (Eklund et al. 2016). Of note, no cluster-extent threshold was used in addition to the (FWE-corrected, SVC) voxel peak-level thresholding.

MVPA with empirical chance levels - results
When performing the MVPA analyses with permutation derived empirical chance level maps, we additionally (to results with theoretical chance levels) observed a significant discrimination between the positive and negative conditions in the right anterior insula and VS.

Suppl. Table 2. Multivariate pattern analyses (searchlight decoding) within ROIs with voxel-wise one-sample t-test (against permutation derived empirical chance levels) for the contrast ‘negative vs. positive’ future thinking (n=31; all p: FWE, small-volume corrected).

ROI (bilateral)	L/R	Cluster size	Peak-voxel activity			*p* – peak voxel FWE
			x	y	z	
positive vs. negative						
ventral striatum	R	11	12	14	1	<0.01
vmPFC	-	100	0	41	-11	0.02
PCC	L	241	-6	-46	19	<0.01
insula	L	91	-33	8	13	0.01
	R	30	42	23	4	0.03

vmPFC: ventromedial prefrontal cortex; PCC: posterior cingulate cortex; FWE: family wise error corrected

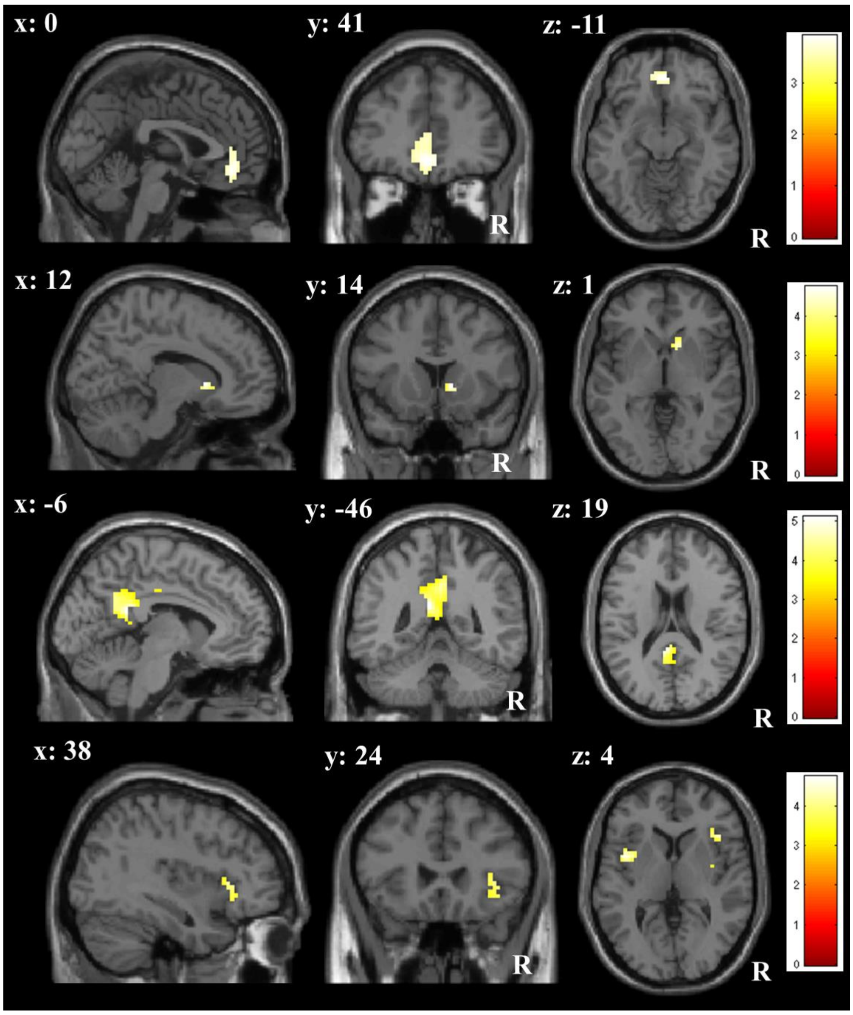

Suppl. Figure 3: Multivariate pattern analyses (searchlight decoding) within ROIs with permutation derived empirical chance levels for the contrast 'negative vs. positive' future thinking revealed significant discriminatory information (all $p<.05$ small-volume FWE-corrected) in the vmPFC, ventral striatum, PCC, and bilateral insula (activation displayed at $p<.001$ uncorrected for illustrative purposes).

References – supplementary material:

Chib VS, Rangel A, Shimojo S, O'Doherty JP. 2009. Evidence for a Common Representation of Decision Values for Dissimilar Goods in Human Ventromedial Prefrontal Cortex. Journal of Neuroscience 29:12315–12320.

Combrisson E, Jerbi K. 2015. Exceeding chance level by chance: The caveat of theoretical chance levels in brain signal classification and statistical assessment of decoding accuracy. Journal of Neuroscience Methods 250:126–136.

Eklund, A., Nichols, T. E., & Knutsson, H. (2016). Cluster failure: Why fMRI inferences for spatial extent have inflated false-positive rates. Proceedings of the National Academy of Sciences of the United States of America, 113(28), 7900–7905.

Hare TA, Camerer CF, Rangel A. 2009. Self-control in decision-making involves modulation of the vmPFC valuation system. Science 324:646–648.

Hare TA, Malmaud J, Rangel A. 2011. Focusing Attention on the Health Aspects of Foods Changes Value Signals in vmPFC and Improves Dietary Choice. Journal of Neuroscience 31:11077–11087.

Litt A, Plassmann H, Shiv B, Rangel A. 2011. Dissociating Valuation and Saliency Signals during Decision-Making. Cerebral Cortex 21:95–102.

Ludwig, V.U., Stelzel, C., Krutiak, H., Magrabi, A., Steimke, R., Paschke, L.M., Kathmann, N., Walter, H., 2014. The suggestible brain: posthypnotic effects on value-based decision-making. Soc Cogn Affect Neurosci 9, 1281–1288.

Plassmann H, O'Doherty J, Rangel A. 2007. Orbitofrontal Cortex Encodes Willingness to Pay in Everyday Economic Transactions. Journal of Neuroscience 27:9984–9988.

Plassmann H, O'Doherty JP, Rangel A. 2010. Appetitive and Aversive Goal Values Are Encoded in the Medial Orbitofrontal Cortex at the Time of Decision Making. Journal of Neuroscience 30:10799–10808.

Schubert R, Ritter P, Wustenberg T, Preuschhof C, Curio G, Sommer W, Villringer A. 2008. Spatial Attention Related SEP Amplitude Modulations Covary with BOLD Signal in S1--A Simultaneous EEG--fMRI Study. Cerebral Cortex 18:2686–2700.

Turkeltaub PE, Eden GF, Jones KM, Zeffiro TA. 2002. Meta-analysis of the functional neuroanatomy of single-word reading: method and validation. Neuroimage 16:765–780.
